# High prevalence of myositis in a southeastern United States pediatric systemic lupus erythematosus cohort

**DOI:** 10.1186/1546-0096-9-20

**Published:** 2011-08-09

**Authors:** Jessica L Record, Timothy Beukelman, Randy Q Cron

**Affiliations:** 1 Department of Pediatrics, Division of Rheumatology, University of Alabama at Birmingham, Birmingham, Alabama 35294, USA

**Keywords:** lupus, myositis, pediatric, mixed connective tissue disease, magnetic resonance imaging, prevalence

## Abstract

Inflammatory myositis is reported in 4-16% of adult systemic lupus erythematosus (SLE) patients. The aim of this study was to determine the prevalence of myositis in a cohort of pediatric SLE patients in the southeastern United States. A retrospective chart review was performed of 55 SLE patients evaluated by Pediatric Rheumatologists in Alabama since January 1, 2008. Patients were defined as having myositis if they satisfied one of the following categories: 1) Proximal muscle weakness on exam with lower extremity muscle edema on MRI; 2) Proximal muscle weakness with elevation in CK, AST, aldolase, or LDH muscle enzymes; or 3) Patient reported weakness or muscle pain and an elevated CK. Inflammatory myositis was present as a feature of SLE in 31% (n = 17) with a 95% confidence interval of 19-45%, statistically different from the reported rates of 4-16% (p < 0.0001). Myositis was positively associated with the presence of anti-ribonucleoprotein antibodies (p = 0.009). Negative associations with myositis were the presence of anti-double stranded DNA antibodies (p = 0.02) and hematologic disorders (p = 0.02). Thus, in the state of Alabama, pediatric SLE myositis is present at a statistically higher rate than previously published values of adult SLE myositis, possibly reflecting geographic (genetic or environmental) and/or age-of-onset related influence(s).

## Introduction

Systemic lupus erythematosus (SLE) is a multisystem autoimmune disease that is extremely heterogeneous in both its clinical and serological presentation. SLE has significant overlapping features of other conditions of similar etiology, including mixed connective tissue disease (MCTD) and Sjögren syndrome. In order to distinguish SLE clinically from such entities, the American College of Rheumatology (ACR) has set forth eleven classification criteria (revised in 1997), of which the patient must have four in order to be classified with SLE [1]. The criteria are both highly sensitive and specific for SLE, though there are still significant discrepancies in the literature with regard to classifying these conditions when overlapping symptoms and serologies are present [2,3].

Although commonly thought of as a disease of middle age, nearly 15% of SLE presents in patients under the age of 16. Additionally, the presenting features in children are often more severe than in adults, and these children have been shown to be twice as likely to require higher dose corticosteroids [4-6]. Though not an ACR classification criterion, myositis has traditionally been recognized as a feature in of 4-16% of adult patients with SLE [7-12]. The prevalence of myositis in pediatric patients with SLE is not reported in these studies. In a small percentage of patients, myositis can actually be the predominant presenting feature [9]. Clinical features of myositis include proximal weakness, myalgia, and muscle atrophy [13,14]. Patients with myositis generally have elevated serum creatine kinase (CK) levels, proximal muscle inflammation by MRI, and abnormal muscle biopsies. In patients with juvenile dermatomyositis, 3-10% also have another autoimmune disease, the most common of which is scleroderma [15,16]. In one study, children with overlap myositis syndromes were shown to be milder with lower CK values and better treatment response [17], whereas other data from adult cohorts suggests that myositis overlap is more severe and associated with higher mortality [18].

Symptoms that have been shown to correlate with myositis in SLE patients include Raynaud phenomenon, anemia, alopecia, oral ulcers, erosive joint disease, and Sjögren syndrome [9,18,19]. These patients have also been shown to less likely have lupus nephritis, though this finding was not found to be statistically significant [18]. Immunologically, anti-RNP antibodies have been shown to be more likely in the myositis group [18]. The aim of this study was to determine the prevalence of clinically evident myositis in pediatric lupus patients in a cohort of 55 patients from a single tertiary care center in the state of Alabama and to compare it with the reported prevalence of 4-16%. The widely accepted Bohan and Peter criteria for myositis are dependent upon muscle biopsy and electromyography results, both of which are invasive and painful procedures not routinely conducted in our practice. Therefore, clinical and laboratory definitions of myositis were established. We also determined the demographic, clinical, serological, and laboratory features associated with lupus myositis in this cohort and compare them with established associations in the adult population.

## Materials and methods

### Patient selection

The records of all patients seen after January 1, 2008 at the Children's Hospital of Alabama (CHA) with the ICD-9 code of SLE on their electronic medical records (n = 98) were identified. Of these 98 patients, 25 were excluded because they had not been evaluated by the Division of Pediatric Rheumatology at CHA and did not have sufficient data to assess the presence of muscle involvement, 2 because they had drug induced lupus, and 16 because they either did not satisfy at least 4 out of 11 ACR criteria for the classification of SLE [1] or there was insufficient data available to make such a determination. The 25 patients excluded that were not evaluated by pediatric rheumatology were similar in makeup to the final cohort, except for that they all had renal manifestations of their SLE and were being followed by pediatric nephrologists. The remaining 55 patients were included in these analyses. General demographic data collected included the following: 1) age of onset of the first manifestations of SLE; 2) race/ethnicity, defined as white, black, Hispanic, or Asian; and 3) sex. Electronic medical records were retrospectively reviewed and data was collected in a standard electronic form. Institutional review board approval was obtained prior to data collection.

### Laboratory data

Lab values analyzed were complement component 3 (C3), complement component 4 (C4), total hemolytic complement (CH50), creatine kinase (CK), lactate dehydrogenase (LDH), aldolase, alanine aminotransferase (ALT), aspartate aminotransferase (AST), and γ-glutamyl transpeptidase (GGT). The lowest level of C3, C4, and CH50 from the available laboratory data was recorded, and it was noted whether this value was considered to be low, normal, or high according to the reference range from the laboratory performing the test. For the enzymes analyzed (CK, LDH, aldolase, ALT, AST, and GGT) the highest level from the available data was recorded, and it was similarly noted whether this value was considered low, normal, or high.

### Antibody profile

The presence of the following antibodies was determined: anti-nuclear antibodies (ANA), ANA pattern, anti-Smith antibodies (Sm), anti-double-stranded DNA antibodies (dsDNA), U1-sn ribonucleoprotein antibodies (RNP), scleroderma-70 antibodies (Scl-70), Ro/SS-A, La/SS-B, rheumatoid factor (RF), lupus anticoagulant (LA), β2-glycoprotein I antibodies (IgM and IgG only), and anticardiolipin (aCL) antibodies (IgM and IgG only). Patients were considered positive if their laboratory data showed elevated titers of these antibodies compared to the lab's reference range at any point in time. In addition, it was noted whether the patient had a reactive rapid plasma reagin (RPR), or if a partial thromboplastin time (PTT) was increased on at least two occasions.

### Other SLE features

The presence of any of the 9 ACR SLE clinical diagnostic classification criteria was recorded. In addition, the presence of alopecia, Raynaud phenomena, hypothyroidism, and headaches were documented.

### Definition of myositis

The Bohan and Peter criteria are the gold standard for defining myositis, more specifically dermatomyositis, in the literature. These criteria require muscle biopsy and EMG [20,21] which are invasive and painful procedures not routinely done in our practice, so at best, patients in our cohort could only be described as having probable polymyositis. Additionally, most centers do not use this definition when describing patients with established SLE or MCTD. Therefore, we established clinical and laboratory based definitions for this study. Patient histories were reviewed for complaints of muscle weakness and muscle pain. Physical exam notes were reviewed for muscle strength testing. For the purpose of this study, patients were defined as having myositis if they satisfied one of the following categories at any time during the course of their disease:

1. Proximal muscle weakness on physical exam with evidence of muscle edema on MRI [T2 or Short T1 Inversion Recovery (STIR)] images of the lower extremity

2. Proximal muscle weakness on physical exam with an elevation in one of the following muscle enzymes: CK, AST, aldolase, or LDH

3. Patient reported muscle weakness or muscle pain (without weakness noted on physical exam) and an elevated CK

### Statistical analysis

The proportion of lupus patients with myositis in this cohort was determined and the Clopper-Pearson exact binomial 95% confidence interval was calculated. The prevalence rate of myositis in the cohort was compared to previously published rates of myositis using the chi-square test. Associations between myositis and the presence of clinical and laboratory factors were examined using Fisher's exact test, Wilcoxon rank-sum test, and one-way ANOVA where appropriate. Statistical analyses were performed using STATA 10.0 (StataCorp, College Station, TX, USA).

## Results

The mean age of our cohort at the time of this study was 16 years (range, 9 to 21 years). The mean age at onset of symptoms attributable to SLE was 13 ± 3 years (range, 3 to 18 years), 87.3% were female, and 74.5% were African-American. All 55 patients in the cohort were ANA positive. There was a high prevalence of anti-Sm antibodies in our cohort, with 69% of the entire cohort having a positive Sm antibody.

Myositis was present as a feature of SLE in 31% (n = 17) with a 95% confidence interval of 19 to 45%. In the myositis portion of the cohort, 13 (76%) of were female, 14 (82%) were African-American, and the mean age at initial diagnosis of SLE was 13.1 ± 3.9 years old. This data was not statistically different from the non-myositis portion of the cohort. The mean age of diagnosis of lupus myositis was 14 ± 4 years (range, 8 to 18 years), which was on average 1.4 years after the initial SLE diagnosis with 8 of 17 patients (47%) presenting with myositis at their initial SLE diagnosis. SLE activity at time of myositis diagnosis was assessed by the physician global assessment score which was available for 9 of the 17 myositis patients. For these 9 patients, the average physician global assessment score was 47 with a range of 8 to 70, with < 5 being correlating with disease remission and 100 being the maximum amount of disease activity. The proportion of children with myositis in our cohort was significantly higher than each of the 6 previously reported SLE cohorts [7-12] (p < 0.05 for all comparisons, ranging from p < 0.0001 to p < 0.047). If all patients from these previously reported cohorts are combined, the resultant prevalence of 7.6% (95% CI 6.1%-9.2%) is much lower than was present in our cohort (p < 0.0001). Of these patients with myositis, 2 patients had weakness on physical exam and evidence of muscle of edema on MRI, 12 patients had weakness on physical exam with an elevation in CK, AST, aldolase, and/or LDH, and 3 patients had self-reported muscle pain or weakness with an elevated CK. If the 3 patients in the latter category with subjective muscle pain or weakness are excluded, the rate of myositis in our SLE cohort becomes 26.9% which remains statistically higher than the reported rate of 4-16%. Only 2 patients in the cohort had MRI testing conducted. With the exception of 1 patient in the cohort, their muscular symptoms were typically mild. This cohort of pediatric SLE patients all met formal SLE criteria but those with myositis shared features with MCTD, with 3 of the lupus myositis patients meeting the Kusukawa criteria (RNP, Raynaud or swollen fingers, and 1 or more findings consistent with lupus, scleroderma, and/or polymyositis) for MCTD [3], and none of the non-myositis cohort met these MCTD criteria. Moreover, if the three patients meeting MCTD criteria are excluded from the analysis, then 26.9% of our SLE cohort had myositis, which remains statistically higher than the reported rate of 4-16%.

The high prevalence of myositis among this pediatric SLE cohort suggested there may be unique disease features associated with the presence of myositis. There were no obvious statistically significant demographic differences in those with and without myositis, at least at the level of statistical power afforded by the size of the patient cohort.

Next, the clinical SLE features in the myositis and non-myositis groups were compared and are summarized in Table 1. Skin involvement (malar or discoid rash) and hematologic disorders occurred less frequently in the myositis group; of these, only the negative association with hematologic disorders (Coombs positive hemolytic anemia, thrombocytopenia, neutropenia, or lymphopenia) was determined to be statistically significant (p = 0.02).

**Table 1:** Clinical features in pediatric SLE according to myositis status

	Myositis group, *n *(%)		Non-myositis group, *n *(%)		Entire cohort, *n *(%)	
Malar rash	5	(29)	19	(50)	24	(44)
Discoid rash	1	(5.9)	8	(21)	9	(16)
Photosensitivity	3	(18)	8	(21)	11	(20)
Oral ulcers	9	(53)	17	(45)	26	(47)
Arthritis	11	(65)	26	(68)	37	(67)
Serositis	6	(35)	10	(26)	16	(29)
Renal disorder	8	(47)	25	(66)	33	(60)
Neurological disorder	1	(5.9)	5	(13)	6	(11)
**Hematologic disorder^a^**	**5**	**(29)**	**25**	**(66)**	**30**	**(55)**
Alopecia	5	(29)	9	(24)	14	(25)
Raynaud phenomenon	5	(29)	12	(32)	17	(31)
Hypothyroidism	1	(5.9)	3	(7.9)	4	(7.3)

Myositis group, *n *= 17; non-myositis group, *n *= 38; entire cohort, *n *= 55. ^a^Statistically significant inverse association with myositis (p = 0.02).

In addition to clinical criteria, immunologic criteria were compared between the myositis and non-myositis subgroups of SLE patients. The main hematologic and immunologic features of SLE in the myositis and non-myositis group are summarized in Table 2. Patients with myositis were more likely to be RNP (p = 0.009) and Sm (p = 0.06) positive, and less likely to be dsDNA positive (p = 0.02). Additionally, although not statistically significant, myositis patients were less likely to have hypocomplementemia (low C3, C4 or CH50) which correlates with the finding that they were also less likely to have renal involvement (Table 1). Some of the immunologic associations with myositis (presence of RNP and speckled ANA pattern and absence of dsDNA) were more reminiscent of MCTD than SLE. Thus, although all the patients met formal ACR classification criteria for SLE, those with myositis shared features of MCTD.

**Table 2:** Immunologic and hematologic features in pediatric SLE according to myositis status

	Myositis group, *n *(%)		Non-myositis group, *n *(%)		Entire cohort, *n *(%)	
ANA	17	(100)	38	(100)	55	(100)
*ANA pattern:*						
Speckled	11	(65)	19	(50)	30	(55)
Homogeneous	5	(29)	16	(42)	21	(38)
Nucleolar	1	(5.9)	0	(0)	1	(1.8)
Dual	0	(0)	3	(7.9)	3	(5.5)
**Sm^a^**	**15**	**(88)**	**21/35**	**(60)**	**36/52**	**(69)**
**dsDNA^b^**	**10**	**(59)**	**34**	**(89)**	**44**	**(80)**
**RNP^c^**	**16**	**(94)**	**20/35**	**(57)**	**36/52**	**(69)**
Scl-70	0/16	(0)	1/27	(3.7)	1/43	(2.3)
Ro/SS-A	8	(47)	16/32	(50)	24/49	(49)
La/SS-B	5	(29)	7/32	(22)	12/49	(24)
RF	3/9	(33)	6/21	(29)	9/30	(30)
Low C3	4	(24)	15	(39)	19	(35)
Low C4	7	(41)	24	(63)	31	(56)
Low CH50	2/5	(40)	12/20	(60)	14/25	(56)
β2-glycoprotein I	3/14	(21)	4/30	(13)	7/44	(16)
aCL (IgG and/or IgM)	3/14	(21)	17/29	(59)	20/43	(47)
LA	2/13	(15)	7/32	(22)	9/45	(20)
Increased PTT	5/14	(36)	6/32	(19)	11/46	(24)
Reactive RPR	2/6	(33)	4/11	(36)	6/17	(35)

Myositis group, *n *= 17; non-myositis group, *n *= 38; entire cohort, *n *= 55. ^a^Near statistically significant direct association with myositis (p = 0.06). ^b^Statistically significant inverse association with myositis (p = 0.02). ^c^Statistically significant direct association with myositis (p = 0.009).

## Discussion

This study analyzed the prevalence and associated characteristics of inflammatory myositis in a cohort of SLE patients from a single center in the state of Alabama that follows all cases diagnosed within a large geographic referral area. All patients met ≥ 4 of the 1997 revised ACR criteria for SLE classification. Due to lack of invasive testing, patients only met possible Bohan and Peter myositis criteria, and, furthermore, these criteria are not typically applied to patients with previously diagnosed SLE or MCTD in the literature. Therefore, the authors felt it was more appropriate to designate clinical and laboratory parameters for defining myositis in this study, though this classification is not established nor validated in the medical literature.

Interestingly, there was a surprisingly high prevalence of clinical myositis (31%) among this lupus cohort. By comparison, Font et al. reported 39 of 600 (6.5%) adult SLE patients with myositis as a component, which was shown to have statistically significant association with the presence of Raynaud phenomenon, anemia, and anti-RNP when compared to patients without muscle involvement [9]. Similarly, Estes and Christian reported 7 of 150 (5%) adult SLE patients with inflammatory myositis [8], and Feinglass et al. reported muscle involvement in 15 of 140 (11%) adult SLE patients [7]; neither publication determined any symptoms associated with the presence of myositis. Tsokos et al. reported that 18 of 228 (8%) analyzed had significant muscle involvement; however, of the 11 with muscle biopsies, none met histological criteria for inflammation [11]. Michet et al. demonstrated 2 patients with features of SLE and polymyositis and 2 patients with features of SLE, polymyositis, and scleroderma out of a cohort of 46 patients (9%) that included both patients with definite and probable SLE [12]. Fessel et al. showed 10 of 64 (16%) to have polymyositis, which was typically clinically mild, but they did not report any associated symptoms [10]. The proportion of children with myositis in our cohort was significantly higher than all prior reports. Unpublished data from the 641 adult SLE patients in the LUMINA cohort demonstrated 103 (16.1%) with myositis/muscle weakness attributable to lupus. Muscle enzymes, EMGs or biopsies were not specifically reported (personal communication, Graciela Alarcon, University of Alabama at Birmingham, July 14, 2011). These previously reported rates used for comparison, however, are all based on analysis of adult cohorts, whereas our study is of a pediatric cohort with the average age of onset of first symptoms attributable to SLE being 13 ± 3 years. Because no formal evaluation and reporting of the prevalence of myositis in pediatric SLE have been published previously, it is not currently possible to perform a direct comparison with another geographical area. However, anecdotally, the pediatric rheumatologists involved in this study (TB and RQC) do not recall such a high proportion of pediatric SLE patients having inflammatory myositis in northern California, the state of Washington, or the Philadelphia, PA area.

It is important to note that muscle symptoms are often attributed to accompanying steroid and antimalarial therapy, and an invasive muscle biopsy is the only way to formally rule out this etiology. Though this modality is certainly a possibility in our cohort, this is much less common than inflammatory myositis in diseases like juvenile dermatomyositis. Additionally, the lupus patients' abnormal muscle enzymes appeared to improve with immunosuppressive therapy (including corticosteroids) which argues for inflammatory myopathy and against drug-induced myopathy as the etiology of their clinical symptoms.

It is interesting to note the significant overlapping features the SLE myositis group has with MCTD. All but one myositis patient was RNP positive, and 11 of 17 (65%) had a speckled ANA pattern, though the latter association was not found to be statistically significant. Both of these findings are strongly associated with MCTD. There has been much debate in the literature regarding classifying MCTD, with some even questioning whether it is actually a separate entity or just the early presentation of a yet undifferentiated collagen vascular disease [2,22,23]. There was a strong tendency in our cohort for patients to be both RNP and Sm positive: of the 36 RNP positive patients, 94% (34) were also Sm positive, and of the 36 Sm positive patients, 94% (34) were also RNP positive. This may reflect the nature of the immunoassay used to detect the individual components of the Sm/RNP complex and adds to the complexity of distinguishing SLE from MCTD. In the context of other research regarding associated features of myositis overlap, our findings of the tendency of myositis overlap patients to have anti-RNP positive, renal sparing SLE is consistent with the current literature [18]. Additionally, the rate of anti-Sm antibodies in our cohort was 69%, which is quite high compared to the reported prevalence of 30% in North America [24]. This could be secondary to the fact that our population is largely African-American, and the literature demonstrates that anti-Sm antibodies are known to be more prevalent in African-Americans [24-26].

Our patients were less likely to have hematological involvement, which is in contrast to the associated anemia demonstrated by Font et al. [9]. Other associations demonstrated by Dayal et al. were alopecia, oral ulcers, erosive joint disease, and Sjögren syndrome [18]; these were not demonstrated in our pediatric cohort.

In summary, in the state of Alabama, pediatric SLE myositis is statistically more prevalent than previously published values of adult SLE myositis. The positive association of myositis with anti-RNP antibodies (as a feature of MCTD) is consistent with the medical literature [27]. The presence of both anti-RNP and anti-Sm antibodies further highlights the significant overlap SLE has with other autoimmune diseases such as MCTD and the challenges that come with appropriately classifying and treating these overlap patients. Clinically, myositis can serve as a warning sign for a potential SLE flare and a marker of the effectiveness of treatment. Awareness of myositis in SLE is important for disease management, and based on the current study, myositis, particularly in subtle forms (e.g., low level inflammation as noted by serum enzymes), may be much more common in SLE than previously noted.

## Competing interests

The authors declare that they have no competing interests.

## Authors' contributions

JLR participated in data collection and writing of the manuscript. TB performed the statistical analyses and participated in writing the manuscript. RQC conceived the project, participated in writing the manuscript and is responsible for its final editing. All authors read and approved the final manuscript.
